# Influence of GluN2 subunit identity on NMDA receptor function

**DOI:** 10.1016/j.neuropharm.2013.01.016

**Published:** 2013-11

**Authors:** D.J.A. Wyllie, M.R. Livesey, G.E. Hardingham

**Affiliations:** Centre for Integrative Physiology, School of Biomedical Sciences, University of Edinburgh, Hugh Robson Building, George Square, Edinburgh EH8 9XD, UK

**Keywords:** NMDA receptor, Glutamate receptor, Structure, Biophysics, Pharmacology, Plasticity, Excitoxicity

## Abstract

*N*-methyl-d-aspartate receptors (NMDARs) are ligand-gated ion channels (‘ionotropic’ receptors) activated by the major excitatory neurotransmitter, l-glutamate. While the term ‘the NMDAR’ is often used it obscures the fact that this class of receptor contains within it members whose properties are as different as they are similar. This heterogeneity was evident from early electrophysiological, pharmacological and biochemical assessments of the functional properties of NMDARs and while the molecular basis of this heterogeneity has taken many years to elucidate, it indicated from the outset that the diversity of NMDAR phenotypes could allow this receptor family to subserve a variety of functions in the mammalian central nervous system. In this review we highlight some recent studies that have identified structural elements within GluN2 subunits that contribute to the heterogeneous biophysical properties of NMDARs, consider why some recently described novel pharmacological tools may permit better identification of native NMDAR subtypes, examine the evidence that NMDAR subtypes differentially contribute to the induction of long-term potentiation and long-term depression and discuss how through the use of chimeric proteins additional insights have been obtained that account for NMDAR subtype-dependency of physiological and pathophysiological signalling.

This article is part of the Special Issue entitled ‘Glutamate Receptor-Dependent Synaptic Plasticity’.

## Highlights

► Reviews the control of NMDAR functional properties by GluN2 subunits. ► Assesses structural elements of GluN2 subunits that determine NMDAR heterogeneity. ► Considers the utility of orthosteric and allosteric ligands acting at NMDARs. ► Potential of the NMDAR C-terminal domain as a novel therapeutic target is discussed.

## 1 Introduction

*N*-methyl-d-aspartate receptors (NMDARs) are a subclass of ionotropic glutamate receptors (iGluRs) that play pivotal physiological and pathophysiological roles in the mammalian central nervous system (CNS), the majority of which are tetrameric assemblies of two GluN1 and two GluN2 subunits (for recent reviews see Paoletti (2011), Traynelis et al. (2010)). Classically, glutamate-mediated NMDAR activity, as opposed to AMPA/kainate or metabotropic activity, is identified pharmacologically by its sensitivity to the selective antagonists such a 2-amino-5-phosphonopentanoic acid (AP5; Davies and Watkins, 1982; Evans et al., 1982) or MK-801 (Wong et al., 1986). Uniquely among iGluRs, NMDARs require binding of two different agonists – glutamate and glycine (or d-serine) for their activation. The obligate GluN1 subunit harbours the co-agonist glycine (d-serine) binding site and, although being encoded by a single gene, can exist in eight, spatially and temporally regulated, isoforms (GluN1-1a to GluN1-4a, and, GluN1-1b to GluN1-4b) which arise from the alternative RNA splicing of exons 5, 21 and 22 (Laurie and Seeburg, 1994; Sugihara et al., 1992). Four types of GluN2 subunits exist (A–D) and are encoded by four separate genes. GluN2 subunits contain the glutamate-binding site (Anson et al., 1998; Laube et al., 1997) and display a developmentally and spatially regulated expression pattern (Monyer et al., 1994; Watanabe et al., 1992). In rodents, GluN2B expression is prominent during embryonic and early post-natal development and remains high in the adult brain. Strong GluN2A expression begins around the second post-natal week of development and together with GluN2B subunits these are the predominantly expressed NMDAR subunits in the adult forebrain. Expression of GluN2C and GluN2D subunits is considerably more restricted – GluN2C is expressed highly, albeit not exclusively in the cerebellum, whereas GluN2D expression is highest during early development in the diencephalon, cerebellum and brain stem although, as discussed below, functional GluN2D-containing NMDARs are present at older ages, particularly in the basal ganglia.

The specific sub-cellular localisation of GluN2A and GluN2B-containing NMDARs within the adult forebrain has been of considerable interest given the differing roles of synaptic and extrasynaptic NMDARs in signalling to neuronal survival and death (reviewed in Hardingham and Bading, 2010). The often-stated notion that GluN2A-containing and GluN2B-containing NMDARs are predominately localised at synaptic and extra-synaptic sites, respectively, is an oversimplification. There is evidence that GluN2B-containing NMDARs are enriched at extrasynaptic sites (Groc et al., 2006; Martel et al., 2009b; Tovar and Westbrook, 1999) but this is not to such an extent that they are absent from synaptic sites even when GluN2A expression is high (Gray et al., 2011; Harris and Pettit, 2007; Rauner and Kohr, 2011; Thomas et al., 2006; Tovar et al., 2009).

**Fig. 1:**
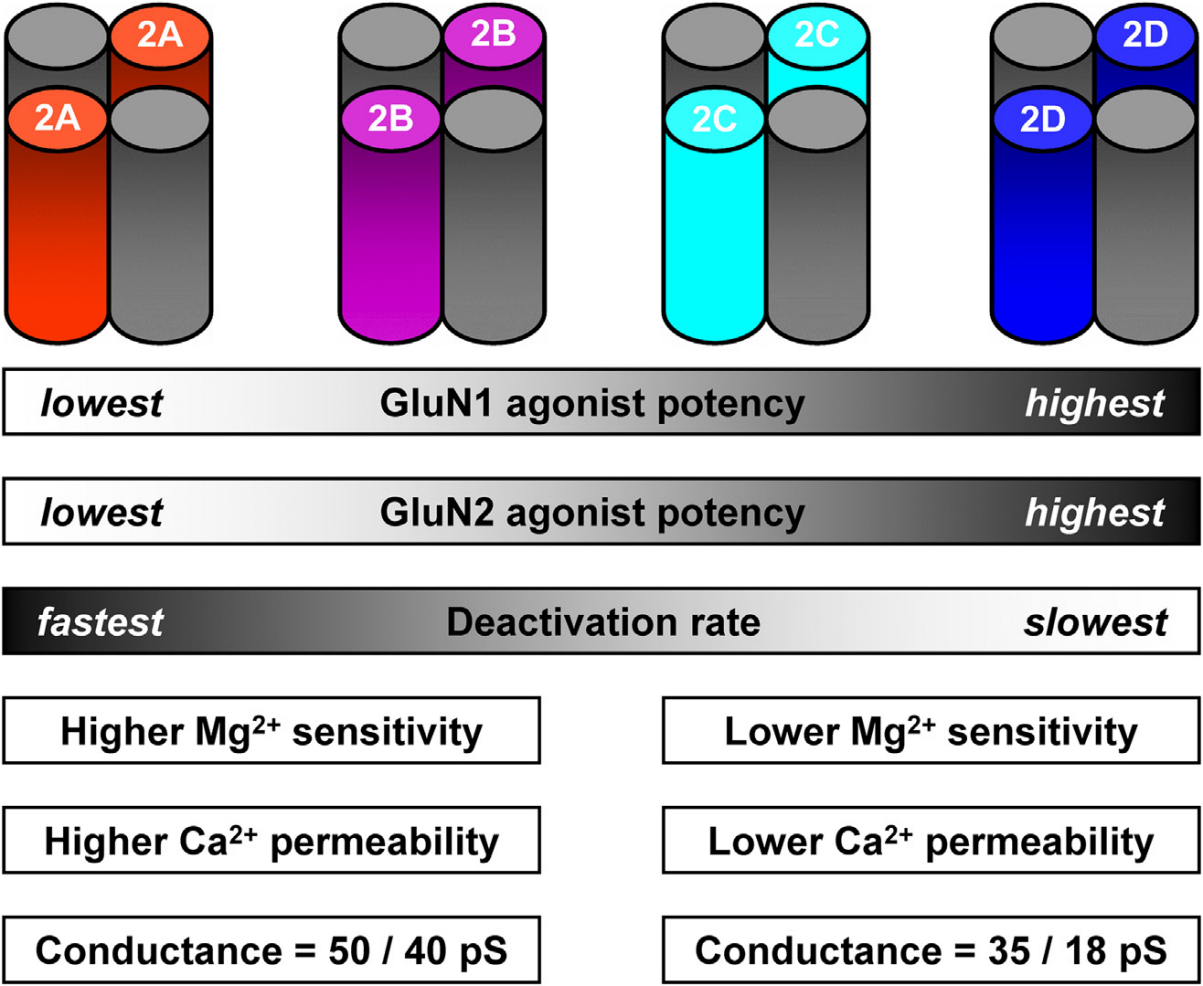
The identity of the GluN2 subunit controls pharmacological and biophysical properties of NMDARs. Schematic representation of each of the four di-heteromeric NMDARs with the GluN1 subunits indicated in grey. Agonist and co-agonist potency is lowest at GluN1/GluN2A NMDARs and highest at GluN1/GluN2D NMDARs. Deactivation rates are fastest for GluN2A-containing NMDARs and slowest for NMDARs containing GluN2D subunits. GluN2B- and GluN2C-containing NMDARs show approximately similar deactivation rates. NMDARs fall into two categories (GluN2A/B-like and GluN2C/D-like) when considering voltage-dependent block by Mg^2+^, permeability to Ca^2+^ and unitary conductance.

As summarised in Fig. 1, the identity of the GluN2 subunit within a di-heteromeric complex endows NMDARs with a unique set of pharmacological biophysical properties (Paoletti, 2011; Traynelis et al., 2010). Briefly, GluN1/GluN2A and GluN1/GluN2B NMDARs display a higher sensitivity to voltage-dependent Mg^2+^ block, higher Ca^2+^ permeability and higher single-channel conductance than their GluN1/GluN2C and GluN1/GluN2D counterparts (Kuner and Schoepfer, 1996; Schneggenburger, 1996; Stern et al., 1992; Wyllie et al., 1996). The potencies of glutamate, NMDA and other related agonist analogues are dependent on the form of the GluN2 subunit (Erreger et al., 2007), which also exerts indirect control over potency for agonists acting at the GluN1 glycine/d-serine binding site (Chen et al., 2008). Furthermore, the nature of the GluN2 subunit also largely determines the deactivation profile of the response to brief applications of agonist, as occurs during synaptic transmission (Monyer et al., 1992; Vicini et al., 1998; Wyllie et al., 1998). For each of these sets of biophysical properties considerable insights have been made recently which highlight how these subtype-dependent parameters are controlled and regulated.

In addition to GluN2 regulation of NMDAR function, a third type of subunit, GluN3 (Chatterton et al., 2002; Ciabarra et al., 1995; Das et al., 1998; Sucher et al., 1995) can also be incorporated into NMDARs and which results in a modification of their properties. Moreover expression of only GluN1 and GluN3 subunits forms a glycine-gated excitatory receptor. Our understanding of the physiological roles for GluN3-containing NMDARs is considerably less advanced than that for ‘classical’ GluN1-GluN2 NMDARs (Pachernegg et al., 2012) and is beyond the scope of this review, which will focus on the pivotal role GluN2 subunits play in determining the functional properties of NMDARs.

## 2 Structure of NMDARs

**Fig. 2:**
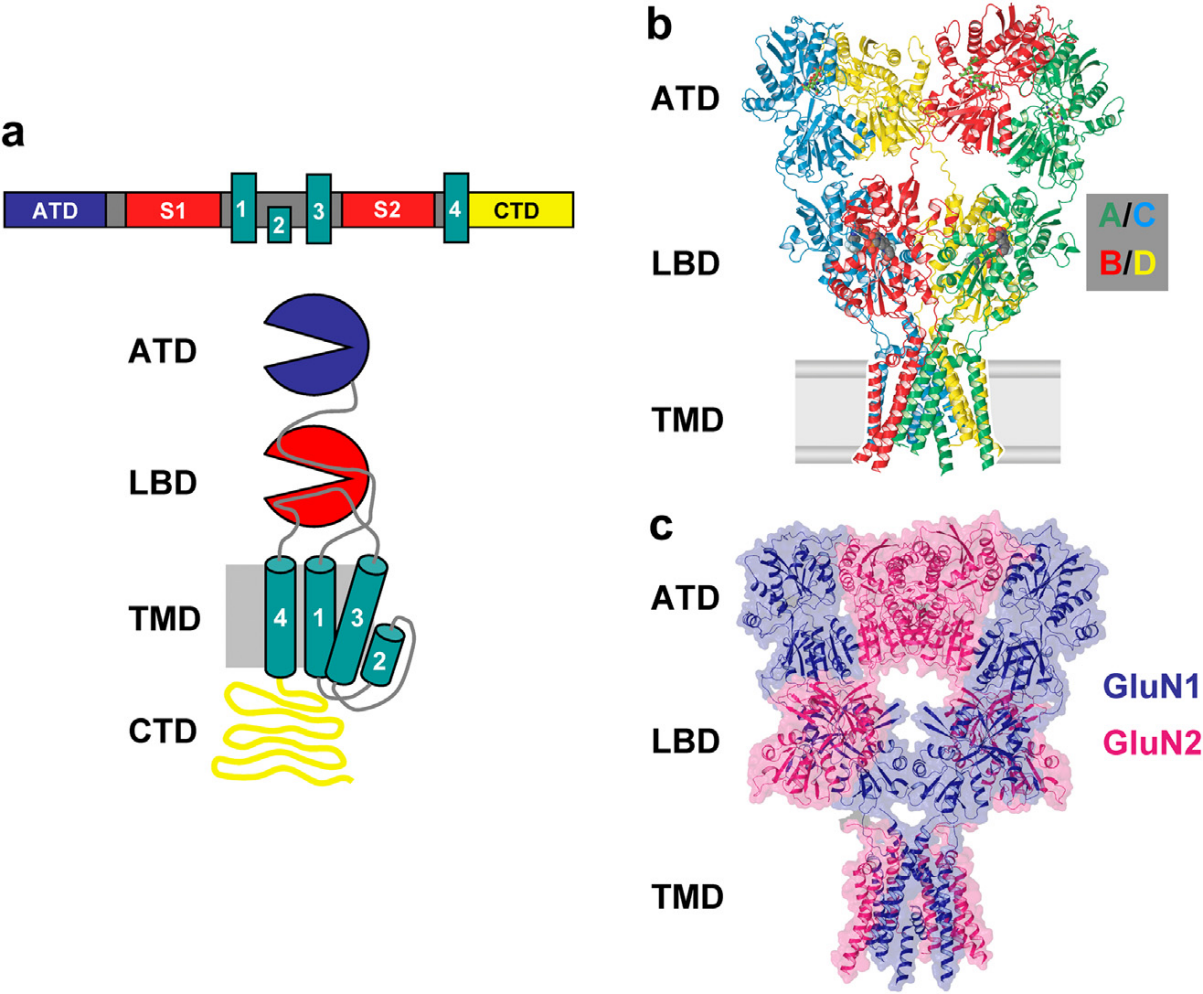
Structure of ionotropic glutamate receptors. (a) Upper panel, linear representation of iGluR subunit highlighting the four functional domains; lower panel, schematic of the general structure of an iGluR subunit indicating the extracellularly located amino terminal domain (ATD) and ligand-binding domain (LBD) the transmembrane domain (TMD) comprised of three membrane-spanning helices (M1, M3 and M4) together with the re-entrant P-loop region of M2 and the large intracellularly located C-terminal domain (CTD). (b) Ribbon structure representation of the rat GluA2 homomeric AMPAR with each of the four subunits coloured coded and indicating the two conformationally distinct pairs of subunits, A/C and B/D. (c) model of the overall architecture of the NMDAR based on the GluA2 crystal structures of the ATD and TMD and the GluN1-GluN2A LBD heterodimer crystal structure. Panels (b) and (c) adapted from Sobolevsky et al. (2009). Copyright © 2009 Nature Publishing Group. Used with permission.

Structural knowledge of vertebrate iGluRs has been fundamentally advanced in recent years by a number of X-ray crystallography studies that describe atomic resolution structures (Kumar and Mayer, 2012). For NMDARs, this includes crystal structures of isolated amino terminal domain (ATD) and ligand-binding domain (LBD) from GluN1 and GluN2 subunits (Farina et al., 2011; Furukawa and Gouaux, 2003; Furukawa et al., 2005; Inanobe et al., 2005; Karakas et al., 2009, 2011), however no complete NMDAR structure has been described to date and therefore the X-ray structure of a closely-related homomeric GluA2 AMPAR provides an invaluable structural surrogate to develop NMDAR models (Sobolevsky et al., 2009). The emerging view of the NMDAR indicates an intricate and domain-specific complexity (Fig. 2).

Initial studies into the quaternary structure of NMDARs proposed a ‘non-alternate’ subunit arrangement (GluN1/GluN1/GluN2/GluN2; Schorge and Colquhoun, 2003), however, experimental evidence from cysteine-linking studies points to an alternating (GluN1/GluN2/GluN1/GluN2) subunit arrangement (Lee and Gouaux, 2011; Riou et al., 2012; Salussolia and Wollmuth, 2012; Sobolevsky et al., 2009) which is further supported by the co-crystallization of GluN1 and GluN2A ATDs in a ‘hetero-dimer’ arrangement (Furukawa et al., 2005).

NMDAR ATD crystals show a bilobar structure comprised of an upper R1 and lower R2 lobe which are formed from the first approximately 380 amino acids of the protein (Farina et al., 2011; Karakas et al., 2009, 2011; Stroebel et al., 2011). Importantly, the NMDAR ATD have very little sequence homology with other iGluR ATDs and notable differences are present between crystal structures of AMPAR and NMDAR ATDs (Furukawa, 2012), the major being the relative position of the R1 and R2 lobes which are twisted by 40–50° with respect to their AMPAR counterparts (Karakas et al., 2009; Stroebel et al., 2011). The ATD harbours allosteric binding sites for a number of ligands such as ifenprodil (and related compounds), protons and Zn^2+^ (Karakas et al., 2009, 2011; Furukawa, 2012). The GluN2B ATD crystal structure reveals the Zn^2+^ binding site to be within the cleft created by the R1 and R2 lobes (Karakas et al., 2009). Conversely, allosteric ligands, such as ifenprodil and Ro25-6981 bind at the dimer interface of GluN1 and GluN2B subunits (Karakas et al., 2011).

The LBD is highly conserved in all GluN2 subunits; indeed, the near equal sequence identity of the glutamate binding site (Chen and Wyllie, 2006) has meant the development of GluN2-selective competitive antagonists has, so far, been largely unrewarding (see below). X-ray crystallography studies of the LBD have been performed with various agonists and competitive antagonists and demonstrate the recognition site is composed of two discontinuous lobes, D1 and D2, also arranged in a bilobar structure (Furukawa and Gouaux, 2003; Furukawa et al., 2005; Inanobe et al., 2005). The D1 region is largely formed from the S1 sequence stretching from the ATD to the start of the first transmembrane domain (M1), while the D2 region is mainly formed by the S2 region located between M3-M4. The crystal structure of the homomeric AMPAR (Fig. 2b) depicts a ‘dimer of dimers’ arrangement at the LBD, however two different subunit conformations exist referred to as A/C and B/D pairs (Sobolevsky et al., 2009). For NMDARs, assuming the ‘alternating’ arrangement GluN1 and GluN2 subunits the A/C pairs would be comprised of GluN1 subunits while B/D pairs would be GluN2 (Sobolevsky et al., 2009).

The transmembrane domain (TMD) represents the most highly conserved portion of NMDAR subunits and consists of three transmembrane helical segments (M1, M3 and M4) with an additional a short re-entrant ‘P’-loop (M2) between the M1 and M3 segments. The crystal structure of the closed AMPAR pore demonstrates that the M3 helices interact close to the iGluR-conserved ‘SYTANLAAF’ motif at the extracellular side of the membrane to form a constriction that forms the channel gate (Sobolevsky et al., 2009). For the open channel configuration, substituted cysteine accessibility studies (Beck et al., 1999; Kuner et al., 1996) indicate that specific portions of the M1, M3 and M4 helices contribute to form a large extracellular vestibule located above the central M2 segment, which forms the narrowest part of the channel. The directly channel-facing apex of the M2 loop in iGluRs harbours the Q/R/N site and creates a major rate-limiting determinant of single-channel conductance, Ca^2+^ permeation and voltage-dependent Mg^2+^ block (Burnashev et al., 1992; Mori et al., 1992; Sakurada et al., 1993; Wollmuth et al., 1998).

The large (>600 amino acids in the GluN2A and GluN2B) intracellularly located CTD is least conserved between GluN2 (Ryan et al., 2008; Traynelis et al., 2010). The CTD contains specific binding motifs for intracellular trafficking and signalling proteins, scaffold proteins and several phosphorylation sites (Lau and Zukin, 2007; Salter and Kalia, 2004). No direct structural information is available for the NMDAR CTD, but is predicted to have a number of α-helical structures as assessed using a bioinformatical approach (Ryan et al., 2008).

## 3 Biophysical properties of NMDAR subtypes

**Fig. 3:**
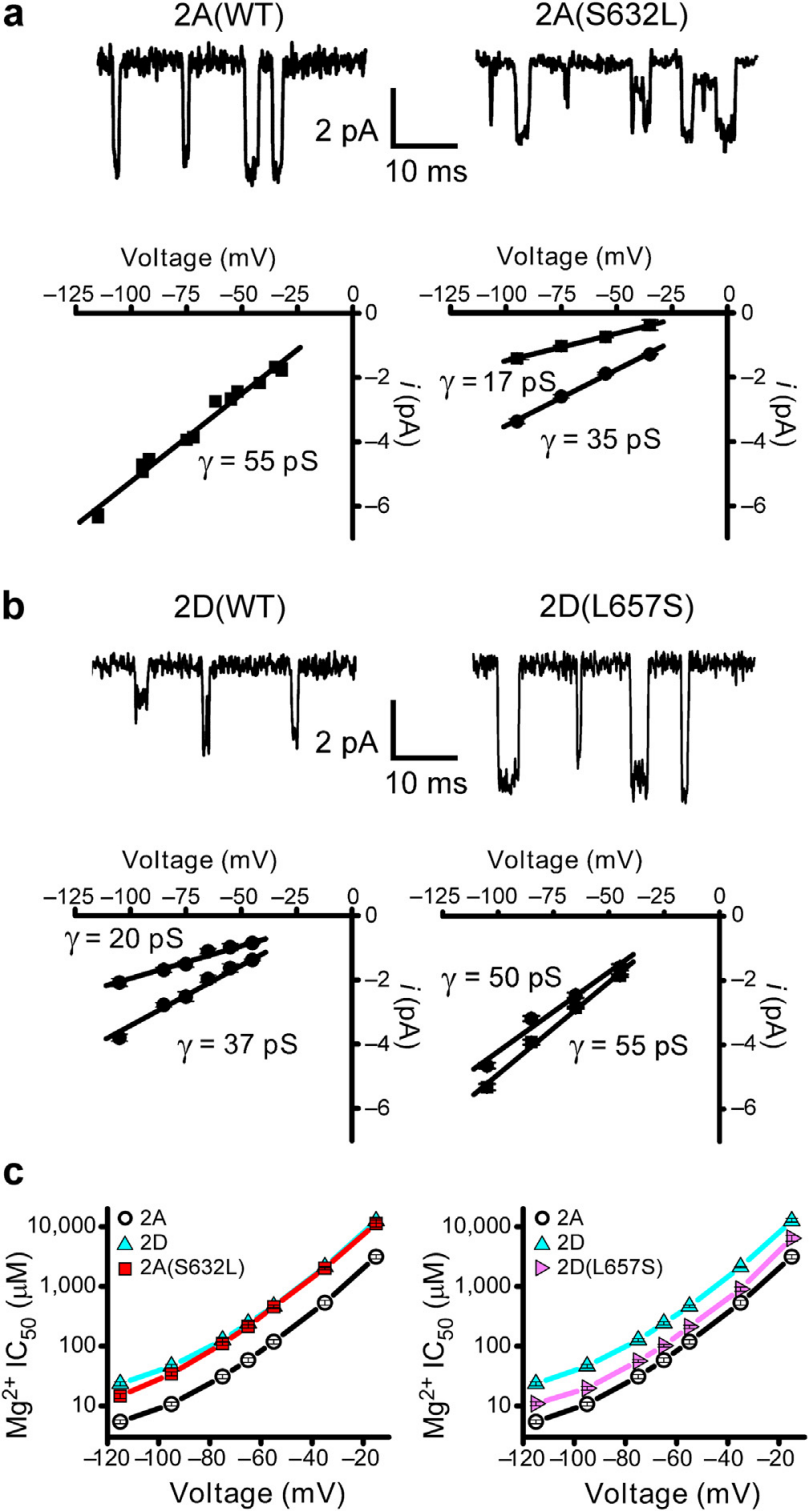
The Ser/Leu site in M3 controls NMDAR single-channel conductance (γ) and voltage-dependent block by Mg^2+^. (a) The characteristic high (∼50 pS) conductance of GluN1/GluN2A NMDARs is changed to a GluN1/GluN2D-like conduction by the point mutation GluN2A(S632L). (b) The reciprocal mutation in GluN2D that replaces the leucine residue at position 657 by serine gives rise to single-channel currents with a GluN1/GluN2A-like conductance. (c) The IC_50_ values for voltage-dependent Mg^2+^-block for GluN1/GluN2A(S632L) are comparable to those for GluN1/GluN2D NMDARs while IC_50_ values for GluN1/GluN2D(L657S) resemble those for GluN1/GluN2A NMDARs. Panels (a)-(c) from Siegler Retchless et al. (2012). Copyright © 2012 Nature Publishing Group. Used with permission.

As stated above the NMDARs fall into two distinct classes when considering their sensitivity to voltage-dependent block by extracellular Mg^2+^, their permeability to Ca^2+^ and their single-channel conductance (Wyllie and Traynelis, 2012). It has been known for many years that each of these properties is considerably affected by mutations of asparagine residues located at the Q/R/N site located near the apex of the M2 re-entrant loop of both GluN1 and GluN2 NMDAR subunits (Burnashev et al., 1992; Mori et al., 1992; Sakurada et al., 1993; Wollmuth et al., 1998). Nevertheless given the sequence identity between all GluN2 NMDAR subunits it is clear that these residues themselves are not responsible for the differences in the permeation properties that are seen between GluN2A/B and GluN2C/D subunits. Studies using chimeric receptors had identified regions within GluN2 subunits that controlled block by Mg^2+^ and single-channel conductance (Kuner and Schoepfer, 1996; O'Leary and Wyllie, 2009) but the recent identification of a single amino acid residue that controls each of these three signature properties of NMDARs has somewhat simplified our understanding of NMDAR function (Siegler Retchless et al., 2012). Near the intracellular side of the third membrane-associated domain GluN2A/B subunits contain a conserved serine residue (Ser632 in GluN2A) whereas the equivalent position in GluN2C/D subunits contains a leucine residue (Leu657 in GluN2D). Fig. 3 illustrates that the point mutation GluN2A(S632L) converts single-channel conductance from GluN2A/B-like to GluN2C/D-like (Fig. 3a) while the corresponding GluN2D(L657S) mutation converts from GluN2C/D-like to GluN2A/B-like (Fig. 3b). Additionally these pairs of point mutations also convert voltage-dependent Mg^2+^-block and Ca^2+^ permeability such that it now resembles that seen for subunits normally containing either the serine or leucine residue (Fig. 3c). Thus, while the intricate details of the kinetic features of NMDAR function show considerable complexity (discussed next) this finding elegantly demonstrates that there is a single molecular determinant for these aspects of ion permeation and block in NMDARs.

**Fig. 4:**
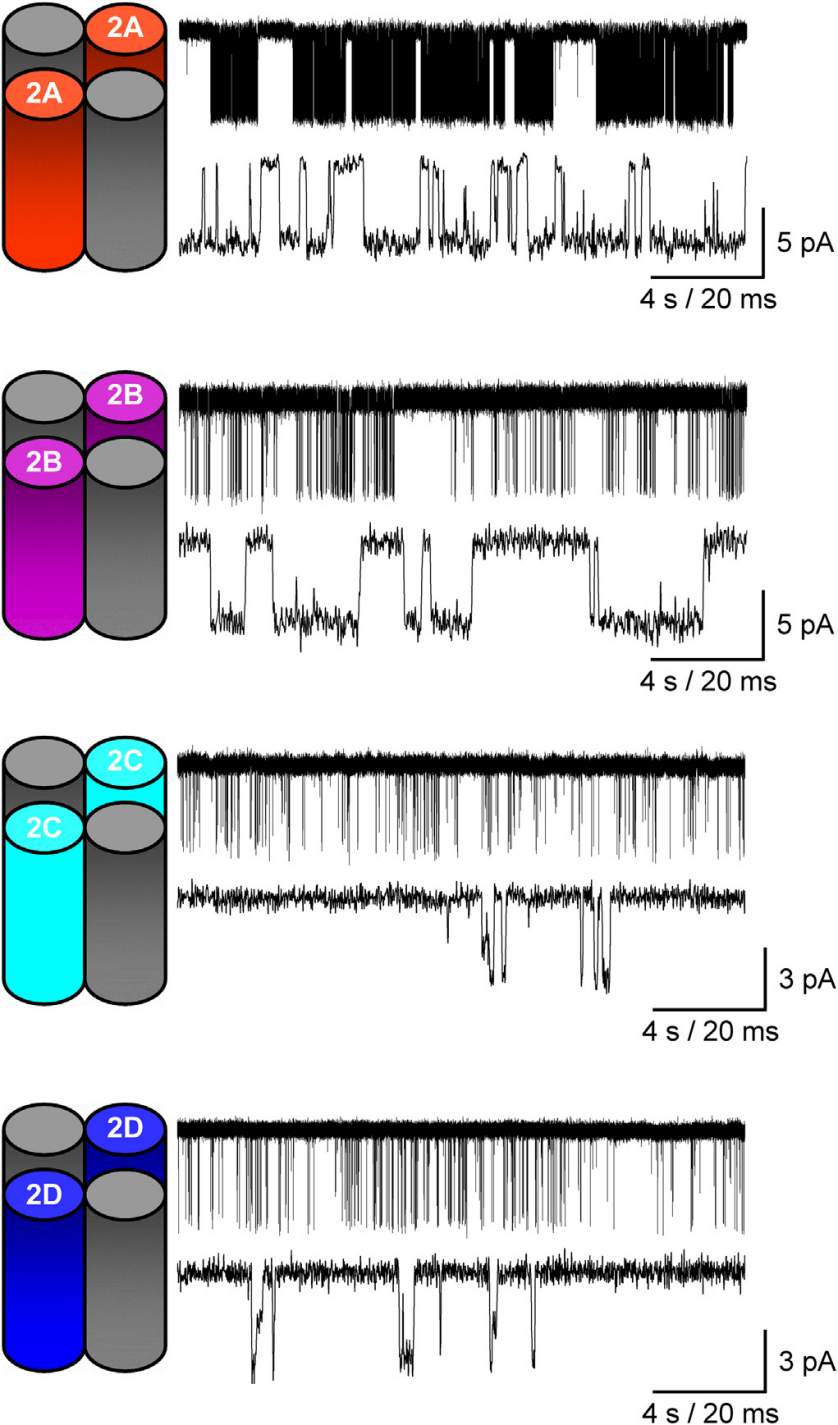
The single-channel characteristics of NMDARs are determined by the GluN2 subunit assembled within the receptor. Steady-state single-channel recordings from excised outside-out membrane patches isolated from HEK293 cells expressing either GluN1/GluN2A, GluN1/GluN2B, GluN1/GluN2C, or GluN1/GluN2D NMDARs. For each receptor combination the upper panel illustrates 20 s of activity while the lower panel shows a selected higher resolution 100 ms period of activity. For all recordings, channel activity was elicited by glutamate (1 mM) in the presence of glycine (0.05 mM) and performed at pH 8.0 in 0.5 mM Ca^2+^. Unpublished data, kindly provided by S. M. Dravid, K. Erreger, K. Ogden, K. M. Vance, and S. F. Traynelis.

The slow deactivation of the NMDAR component of the glutamatergic EPSC is determined by the nature of the underlying individual channel activations which summate and give rise to the macroscopic current while the variations in the time-course of synaptic currents mediated by different NMDAR subtypes are explained by the subtype-dependent differences in the durations for which glutamate remains bound at its binding site. Heterogeneity of activations in single-channel data records are well documented and arise not only because of differences in NMDAR subunit composition but also occur because both native and recombinant NMDARs display modal gating where the activity of a single receptor subtype switches between periods of high, medium and low open probabilities (Amico-Ruvio and Popescu, 2010; Borschel et al., 2012; Popescu and Auerbach, 2003; Popescu et al., 2004; Zhang et al., 2008 and reviewed in Popescu (2012)). Modal gating aside, it is the GluN2 composition of NMDARs that is the main determinant of the nature and duration of single-channel activations of NMDARs. Fig. 4 illustrates single-channel activity for each of the four di-heteromeric GluN1-GluN2 NMDAR subtypes recorded in the presence of saturating concentrations of glutamate (and glycine) and highlights the differences in the behaviour of NMDAR subtypes. GluN1/GluN2A NMDARs possess the highest open probability (around 0.5) while for GluN1/GluN2B NMDARs this parameter is about 3–5 fold lower. These values however are considerably greater than the open probabilities for GluN1/GluN2C and GluN1/GluN2D NMDARs (Amico-Ruvio and Popescu, 2010; Banke and Traynelis, 2003; Chen et al., 1999; Dravid et al., 2008; Erreger et al., 2005a; Popescu and Auerbach, 2003; Popescu et al., 2004; Schorge et al., 2005; Vance et al., 2012; Wyllie et al., 1998; Zhang et al., 2008). Indeed the open probability of GluN2C- and GluN2D-containing NMDARs is exceptionally low (around 0.01–0.04) indicating that even when fully liganded these channels remain in closed states for the vast majority of their activations (Dravid et al., 2008; Vance et al., 2012; Wyllie et al., 1998).

The single-channel records in Fig. 3 illustrate steady-state activity of NMDARs, however synaptic activation of NMDARs occurs under non-equilibrium conditions and therefore in terms of channel activity we need to understand the nature of an individual channel activation that begins with the first opening following agonist binding and ends with the last closing before dissociation of agonist, as would occur during the synaptic release of glutamate. These individual activations or ‘bursts’ of activity, if correctly identified in single-channel recordings, will predict the macroscopic response (Colquhoun et al., 1997). For NMDARs it has long been recognised that the nature of these bursts is exceedingly complex (Gibb and Colquhoun, 1991, 1992; Howe et al., 1991) and contain multiple open and closed states. As is predicted from studies of macroscopic deactivation rates following brief applications of glutamate (Vicini et al., 1998; Wyllie et al., 1998) the duration of these activations is shortest for GluN1/GluN2A NMDARs (30–50 ms) and longest for GluN1/GluN2D NMDARs (2000–4000 ms).

**Fig. 5:**
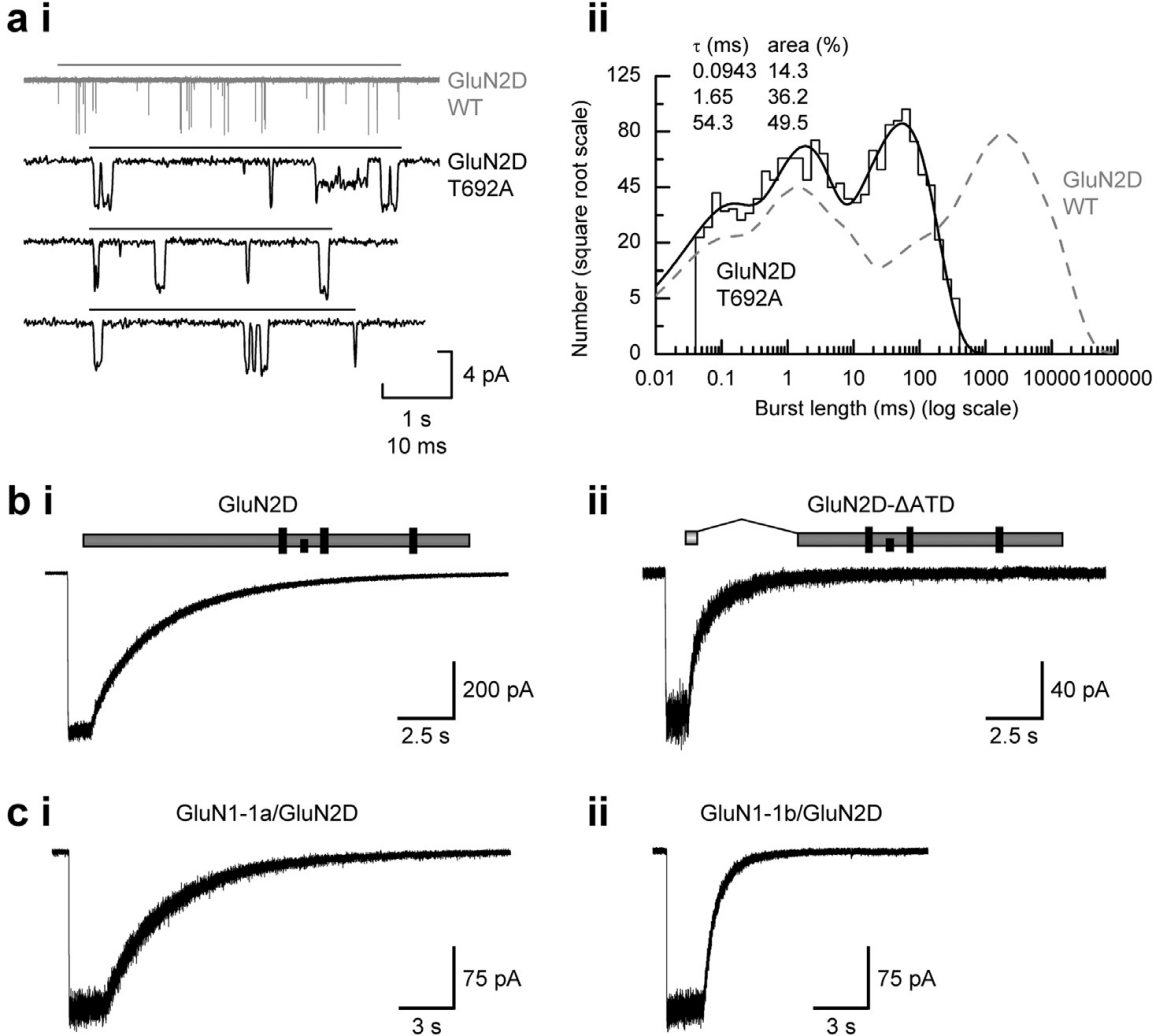
Control of NMDAR deactivation rates. (a) Example of a burst of openings arising from an activation of a wild-type (WT) GluN1/GluN2D NMDAR (ai. upper panel, grey); lower three traces (black) show examples of single activations of GluN1/GluN2D(T692A) NMDARs. For each the line denotes the period between the first opening and last closing of each burst. Comparison of burst length distributions (aii) for GluN1/GluN2D(T692) and WT GluN1/GluN2D NMDARs (shown as a dashed grey line). Note the numbering used to indicate the position of the threonine residue in GluN2D is for the mature protein (i.e. it excludes the signal peptide). (b) Representative whole-cell currents recorded from an HEK293 cell expressing GluN1/GluN2D NMDARs (bi) or GluN1/GluN2D NMDARs in which the ATD was removed (bii). Note the increase in the deactivation rate when the GluN2D ATD is absent. (c) Exon-5 lacking GluN1-1a subunits (ci) when co-expressed with GluN2D form NMDARs with typical slow deactivation rates, whereas GluN1-1b subunits which containing exon 5 (cii) accelerate deactivation of GluN2D-containing NMDARs. Panel (a) from Chen et al. (2004); (b) from Yuan et al. (2009) and (c) from Vance et al. (2012). Used with permission.

While our level of understanding of these events is not as complete as for example nicotinic acetylcholine receptors found at the muscle endplate or inhibitory glycine receptors (Lape et al., 2008; Sivilotti, 2010) kinetic schemes have been proposed for each of the four di-heterometric NMDAR subtypes which describe many of the subtype-specific features of individual activations and accurately describe the time-course of deactivation following brief pulses of agonist application (Amico-Ruvio and Popescu, 2010; Banke and Traynelis, 2003; Dravid et al., 2008; Erreger et al., 2005a; Popescu and Auerbach, 2003; Popescu et al., 2004; Schorge et al., 2005; Vance et al., 2012; Zhang et al., 2008). Point mutations in the GluN2 LBD that increase the rate at which glutamate dissociates from its binding site cause shortening of channel activations and concomitant increases in the rate with which macroscopic currents deactivate following brief applications of agonist (Anson et al., 1998, 2000; Chen et al., 2004). Fig. 5a illustrates the dramatic shortening in GluN1/GluN2D burst lengths when a threonine residue, located in S2 region, is mutated to alanine (Chen et al., 2004). Similarly, agonists with different dissociation rates but acting at the same NMDAR subtype give rise to individual channel activations possessing different mean durations with appropriately altered deactivation kinetics of macroscopic currents (Banke and Traynelis, 2003; Erreger et al., 2005b; Vance et al., 2011). Clearly the rate at which glutamate (or any agonist) dissociates from its binding site determines the duration of the activation. Nevertheless for each GluN2 subunit the residues that H-bond with glutamate in the LBD show few subunit differences (reviewed in Chen and Wyllie (2006)) and of themselves, while being critically involved in contributing to the rate at which dissociation occurs, do not account for the NMDAR subtype-dependence of the duration of channel activations (or the deactivation rates of macroscopic current responses). In this respect two recent studies (Gielen et al., 2009; Yuan et al., 2009) have identified the ATD and the short linker region between the ATD and LBD, together with the LBD itself as structural elements within GluN2 subunits that control NMDAR subtype channel open probability, the duration of channel activations as well as agonist potency (Fig. 5b). Additional regulation of the kinetic behaviours of GluN1/GluN2B and GluN1/GluN2D NMDARs is provided by the nature of the GluN1 splice variant present in the tetrameric receptor assembly (Fig. 5c) (Rumbaugh et al., 2000; Vance et al., 2012). For both of these NMDAR subtypes the presence of exon 5 (the ‘b’ splice variants of GluN1 subunits) causes an increased rate of current deactivation. Indeed if GluN1 ‘b’ splice variants predominate in NMDARs which are thought to be tri-heteromeric assemblies of GluN1 and either GluN2A or GluN2B together with GluN2D (Brothwell et al., 2008; Harney et al., 2008; Logan et al., 2007; Suarez et al., 2010) this may explain why they do not exhibit the very slow decay time-course that is seen when GluN2D subunits assemble with exon-5 lacking (‘a’ forms) of GluN1 (Monyer et al., 1994; Vicini et al., 1998; Wyllie et al., 1998; Yuan et al., 2009).

## 4 GluN2-selective ligands: tools to identify native NMDARs

As discussed above each NMDAR subtype possesses a unique set of biophysical characteristics that allow unambiguous identification of a particular di-heteromeric subunit combination. Nevertheless it is not always possible or desirable to perform such a ‘fingerprint’ analysis of NMDAR properties in order to determine the composition of a population of native NMDARs. Ideally one would want to use ligands which either block or modify NMDAR function in a subtype-selective manner and that would permit the dissection of a native NMDAR population and an elucidation of the role(s) each NMDAR subtype performs. The repertoire of NMDAR subtype-selective antagonists and negative and positive allosteric modulators has been extensively reviewed recently (Monaghan et al., 2012; Ogden and Traynelis, 2011; Paoletti, 2011) and while advances have been made in improving their selectivity there are still relatively few ligands that exist which possess sufficient potency and selectivity to allow unambiguous identification of NMDAR subtypes by pharmacological methods alone. Furthermore, care needs to be taken when working with systems in non-equilibrium conditions, such as synaptic transmission, and comparing data obtained from pharmacological characterisation of parameters that have been determined in steady-state experiments.

For NMDARs comprised of GluN1 and GluN2B subunits the non-competitive, negative allosteric modulators, ifenprodil (Williams, 1993), *R*-(R*,S*)-α-(4-hydroxyphenyl)-β-methyl-4-(phenylmethyl)-1-piperidine propranol (Ro25-6981; Fischer et al., 1997) and (1*S*,2*S*)-1-(4-hydroxyphenyl)-2-(4-hydroxy-4-phenylpiperidino)-1-propanol (CP-101,606; Mott et al., 1998) are exemplars of a wide range of compounds (Mony et al., 2009) that display a selectivity for this receptor combination of around 100-fold over other di-heteromeric NMDAR combinations that allows these antagonists to be used to probe effectively NMDAR subunit composition and function in native neurons. The binding site for these antagonists is located at the interface of the GluN1 and GluN2B ATDs (Karakas et al., 2011) with the determinants of GluN2B susceptibility to block by these antagonists being widely distributed. Indeed only one residue differs between GluN2B (Ile111) and GluN2A (Met112) at the phenylethanolamine binding site and mutation of the GluN2A methionine residue to isoleucine does not confer ifenprodil sensitivity to GluN1/GluN2A NMDARs nor is it lost at GluN1/GluN2B NMDARs if the isoleucine residue is replaced by methionine (Karakas et al., 2011). A recent study (Burger et al., 2012) has mapped extensively the residues at the GluN1-GluN2B ATD dimer interface that control the potency of a large number of GluN2B-selective negative allosteric modulators. Through site-directed mutagenesis and molecular modelling this study demonstrated that there are ligand-specific contacts within this binding for the large number of compounds which act at this allosteric site. This, of course, offers the potential for the further development of drugs with greater potency or which possess less off-target binding. Nevertheless, given the overall near identical amino acid sequence homology between GluN2A and GluN2B at this site, it remains to be determined what structural elements control the very strong subunit-specificity of ifenprodil and related ligands.

The quest for a similarly selective GluN1/GluN2A NMDAR antagonists has been long and has been met with only limited success. While initial reports suggested that (*R*)-[(*S*)-1-(4-bromo-phenyl)-ethylamino]-(2,3-dioxo-1,2,3,4-tetrahydroquinoxalin-5-yl)-methyl]-phosphonic acid (NVP-AAM077) (Auberson et al., 2002) showed 100-fold selectively for human GluN2A-containing NMDARs over GluN2B-containing NMDARs these were later shown not to hold for rodent NMDARs where the difference in *K*_B_ values for NVP-AAM077 acting at the two NMDAR subtypes showed only a 5-fold difference (Frizelle et al., 2006). Residues in the LBD of human and rodent GluN2A and GluN2B subunits differ by only two amino acids between species, neither of which are direct contact residues within the glutamate binding site, and no major differences have been found in other studies that have directly compared other agonist and antagonist potencies at rodent and human NMDARs (Hedegaard et al., 2012; Otton et al., 2011). Indeed the lack of selectively of this antagonist acting at rodent NMDARs was highlighted early on in a study examining synaptic function in GluN2A-lacking mice (Berberich et al., 2005) where NVP-AAM077 at concentrations used to produce what was considered to be selective block of GluN2A-containing NMDARs caused a 60% block of remaining (GluN2B-mediated) synaptic NMDAR current. Nevertheless, NVP-AAM077 continues to be widely used in many studies of NMDAR function and at concentrations where it simply does not discriminate between GluN2A- and GluN2B-containing NMDARs. Furthermore, complications arise when using potent competitive antagonists (such as NVP-AAM077) under non-steady-state conditions as occurs during the process of synaptic transmission. For example, consider an experiment where an antagonist is pre-applied and equilibrium is allowed to be established with the receptor population under investigation. The duration of time available for agonist (neurotransmitter) binding during processes such as synaptic transmission is typically in the order of 1–2 ms and not sufficient for a new receptor/agonist/antagonist equilibrium to be established. Moreover, the lifetime of the receptor–antagonist complexes will outlast considerably the duration that agonist is available for binding and in effect an antagonist that under equilibrium conditions is reversible now behaves in, effectively, an irreversible manner (Wyllie and Chen, 2007). In other words, under the non-stationary kinetics of synaptic transmission the observed potency of the competitive antagonist is increased compared to that determined in an experiment carried out under steady-state conditions and therefore one can easily be misled when comparing the magnitude of antagonism under conditions which are not equivalent.

**Fig. 6:**
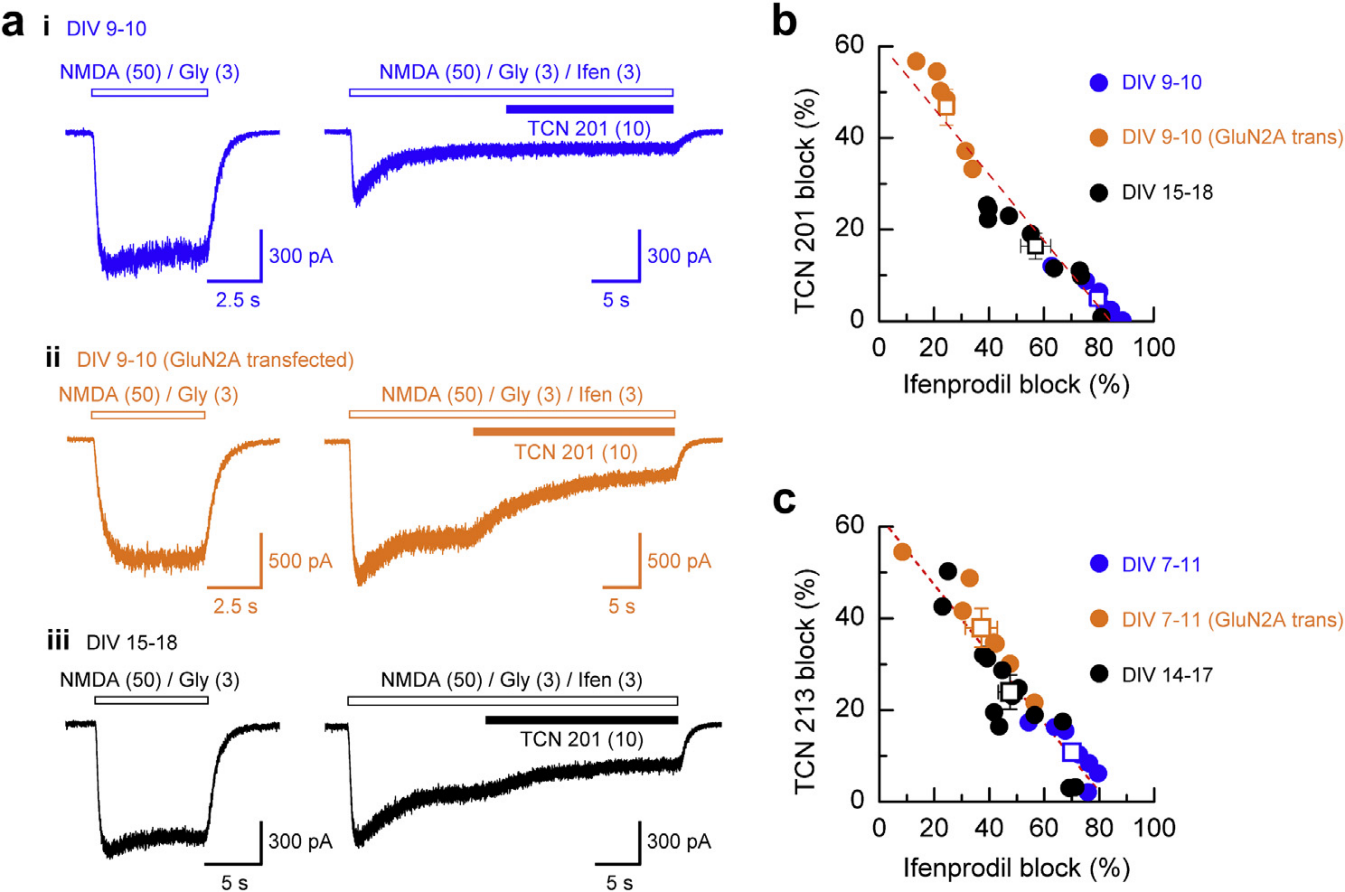
Antagonism of NMDAR-mediated currents by TCN 201. (a) Left, example steady-state whole-cell NMDAR-mediated currents recorded from cortical pyramidal cells from (ai), days *in vitro* (DIV) 9–10 neurones, (aii), DIV 9–10 neurones transfected with GluN2A NMDAR subunits, and (aiii), DIV 15–18 neurones. To the right, traces illustrate the sensitivity of each of these NMDAR-mediated currents to the GluN2B-selective antagonist, ifenprodil and the subsequent sensitivity of the ifenprodil-insensitive component of this current to TCN 201. (b) Plot illustrating the extent of ifenprodil and TCN 201 antagonism of NMDA-evoked currents. Despite a wide range in the amount of block produced by either ifenprodil or TCN 201 the data show a strong (negative) correlation. (c) Equivalent plot to that illustrated in (b) but for antagonism by ifenprodil and TCN 213. Panels (a) and (b) from Edman et al. (2012) and (c) from McKay et al. (2012). Used with permission.

Recently, however, novel GluN2A-selective inhibitors have been identified (Bettini et al., 2010) which show strong selectivity for GluN1/GluN2A NMDARs over GluN1/GluN2B NMDARs. TCN 201 (3-chloro-4-fluoro-*N*-[4-[[2-(phenylcarbonyl)hydrazino]carbonyl]benzyl]benzenesulphonamide) and TCN 213 (*N*-(cyclohexylmethyl)-2-([4-thiadiazol-2-yl]thio)acetamide) each antagonise GluN1/GluN2A NMDAR-mediated currents in a non-competitive but glycine-dependent manner (Edman et al., 2012; Hansen et al., 2012; McKay et al., 2012). In addition, both TCN 201 and TCN 213 block NMDAR-mediated responses in neurones at a developmental time-point where GluN2A subunits are known to be expressed or where neurones have been transfected to over-express GluN2A-containing NMDARs (Edman et al., 2012; McKay et al., 2012) (Fig. 6). The site and mechanism of action of TCN 201 has been examined in detail (Hansen et al., 2012) and it is thought that TCN 201 acts as a negative allosteric modulator of glycine binding by accelerating its dissociation rate. Mutation of residues at the GluN1-GluN2A interface (Leu777, Leu780 and Val783 in GluN2A and Phe754 and Arg755 in GluN1) alters TCN 201 potency and highlight that this interface harbours a novel site for allosteric modulation of NMDARs. Nevertheless, the glycine-dependency of the antagonism afforded by antagonists such as TCN 201 and TCN 213 needs to be taken into account during experimental design. The limited solubility of TCN 201 (the more potent of the two antagonists) means that in situations where glycine (or d-serine) is used at a concentration of 30 μM (to ensure saturation at the GluN1 co-agonist binding site) complete inhibition of a GluN1/GluN2A NMDAR-mediated cannot be achieved (Edman et al., 2012; Hansen et al., 2012; McKay et al., 2012). Furthermore, care needs to be taken to determine the levels of glycine (or d-serine) in experimental preparations so that the expected level of inhibition of GluN1/GluN2A NMDAR-mediated responses can be predicted – this is not always possible in, for example, *in vitro* slice preparations. In addition it should be remembered that many formulations of culture media contain high concentrations of glycine that are considered to be saturating with respect to the NMDAR glycine binding site and therefore the TCN compounds are rendered ineffective under such conditions. Despite these caveats, non-competitive glycine-site negative allosteric modulators such as TCN 201 do not suffer from the same equilibrium/non-equilibrium issues that are highlighted above when using potent competitive glutamate-site antagonists if it is assumed that steady-state conditions are achieved prior to the synaptic glutamate release. The discovery of a novel allosteric binding site in GluN1/GluN2A NMDARs raises the potential for future development of more potent ligands that possess better solubility to allow for the selective and complete block of GluN2A-containing NMDARs.

In addition to ligands which selectively target GluN1/GluN2A and GluN1/GluN2B diheteromeric NMDAR subtypes several novel compounds have been described which selectively inhibit or potentiate NMDARs containing GluN2C or GluN2D subunits. (2R*,3S*)-1-(phenanthrene-3-carbonyl)piperazine-2,3-dicarboxylic acid (UBP141; Morley et al., 2005) shows around 5–10 fold selectively for GluN1/GluN2C or GluN1/GluN2D NMDARs over those containing GluN2A or GluN2B subunits as determined by Schild analysis (Costa et al., 2009). Nevertheless the selectivity of UBP141 is not high and similar issues, to those mentioned above, arise when using such ligands to determine the composition of synaptically located NMDARs containing GluN2C or GluN2D subunits. In this regard the recent discovery of both negative and positive allosteric modulators that act at NMDARs containing GluN2C or GluN2D subunits indicates that as is the case for TCN 201 acting at GluN1/GluN2A NMDARs and ifenprodil acting at GluN1/GluN2B NMDARs that sites out with the glutamate binding site itself offer the best possibility for the selective inhibition (or potentiation) of specific NMDAR subtypes. (*E*)-4-(6-methoxy-2-(3-nitrostyryl)-4-oxoquinazolin-3(4H)-yl)-benzoic acid (QNZ46; Hansen and Traynelis, 2011; Mosley et al., 2010) is a non-competitive antagonist with approximately 50-fold selectivity for GluN2C- and GluN2D-containing NMDARs. Since it requires glutamate binding to the GluN2 subunit (although not glycine binding to GluN1) its blocking action displays use-dependency (Hansen and Traynelis, 2011). Residues which control inhibition by QNZ46 are located in the S2 region of the GluN2 LBD and it is proposed that glutamate occupancy at its binding site either allows accessibility of QNZ46 to its binding site or increases the affinity of the site for QNZ46. 4-(5-(4-bromophenyl)-3-(6-methyl-2-oxo-4-phenyl-1,2-dihydroquinolin-3-yl)-4,5-dihydro-1H-pyrazol-1-yl)-4-oxobutanoic acid (DQP-1105) although structurally unrelated to QNZ46 is also thought to act at this site (Acker et al., 2011). Selective potentiation of GluN2C- and GluN2D-containing NMDARs can be achieved with 3-(chlorophenyl)(6,7-dimethoxy-1-((4-methoxyphenoxy)methyl)-3,4-dihydroiso-quinolin-2(1H)-yl)methanone (CIQ; Mullasseril et al., 2010). In GluN2D a residue in M1 (Thr592) has been identified, potentially, as the site that controls CIQ activity. This residue is conserved in GluN2C but not in either GluN2A or GluN2B subunits. Furthermore, CIQ potentiates NMDAR-mediated currents in neurons from sub-thalamic nuclei (Mullasseril et al., 2010), one of the few brain regions where GluN2D expression is high (Standaert et al., 1994) and which together with neurones from other components of the basal ganglia have been shown to express functional NMDARs containing GluN2D subunits (Brothwell et al., 2008; Jones and Gibb, 2005; Logan et al., 2007; Suarez et al., 2010).

## 5 Tri-heteromeric NMDARs complicate pharmacological studies

The characterisation of the specificity of ligands that block or modify function is, almost always, carried out on recombinantly expressed di-heteromeric NMDARs and then this information is used to probe NMDAR properties in neuronal populations. However, complications arise since native populations are mixed and will be comprised of both di- and tri-heteromeric combinations of GluN1 and GluN2 subunits. Indeed it is considered that in the adult forebrain that a substantial proportion of NMDARs contain together with GluN1 both GluN2A and GluN2B subunits (Chazot and Stephenson, 1997; Rauner and Kohr, 2011). The unique pharmacological properties of tri-heteromeric NMDARs was elegantly demonstrated when it was shown that Zn^2+^ ions and ifenprodil antagonise GluN1/GluN2A/GluN2B NMDARs to a lesser extent than is seen at di-heteromeric GluN1/GluN2A or GluN1/GluN2B NMDARs, respectively (Hatton and Paoletti, 2005). Similarly in early post-natal development an equivalent level of inhibition is observed for NMDAR-mediated currents in forebrain neurones indicative of a predominant GluN1/GluN2B composition of native NMDARs (Brothwell et al., 2008; Carmignoto and Vicini, 1992; Crair and Malenka, 1995; Edman et al., 2012; Flint et al., 1997; Gray et al., 2011; Hestrin, 1992; McKay et al., 2012; Rauner and Kohr, 2011; Sheng et al., 1994; Stocca and Vicini, 1998). At later developmental stages block by ifenprodil, Ro25-6981, CP-101,606 and related allosteric inhibitors decreases, consistent with the increased expression of GluN2A subunits and the presence of a substantial population of native GluN1/GluN2A/GluN2B NMDARs (see for example Edman et al. (2012), Gray et al. (2011), McKay et al. (2012), Rauner and Kohr (2011)). It is also pertinent to note that the presence of extracellular Mg^2+^ decreases the extent of block produced by ifenprodil and CP-101,606 at what are presumed to be native tri-heteromeric GluN1/GluN2A/GluN2B NMDARs (Rauner and Kohr, 2011). This effect of Mg^2+^ is not seen at recombinantly expressed NMDARs nor at native NMDARs composed of predominantly only GluN1 and GluN2B subunits and serves as a reminder that the presence of physiological concentrations of extracellular Mg^2+^ can significantly affect the pharmacological profile of these, together with other commonly used NMDAR antagonists (for example see Kotermanski and Johnson (2009), Otton et al. (2011)).

While we have a good appreciation of the extent of antagonism produced by ifenprodil and related compounds at tri-heteromeric GluN1/GluN2A/GluN2B NMDARs (Hatton and Paoletti, 2005; Rauner and Kohr, 2011) we have a much poorer knowledge of the potency of other antagonists (or potentiators) acting at these or other combinations of tri-heteromeric NMDARs. We do not know, for example, whether the affinity of an antagonist acting at a di-heteromeric GluN1/GluN2 NMDAR is the same when the equivalent GluN2 subunit in now present in a tri-heteromeric complex. While this is possible to test in theory, the difficulty is not with the study of the antagonism *per se* but rather in our ability to generate NMDARs with known subunit combinations. For example, recombinant expression of GluN1 together with two GluN2 subtypes will generate three distinct NMDAR populations. While it is possible to detect using electrophysiological recordings functional NMDARs containing two types of GluN2 subunits (Cheffings and Colquhoun, 2000) controlling the reproducibility of the proportion with which they are expressed with di-heteromeric receptors is more problematic. Assessment of the pharmacological profile of agonists and antagonists, together with the wide range of negative and positive allosteric modulators that are now being identified, at tri-heteromeric NMDARs while challenging, seems to be of critical importance in order to further our appreciation of the functional roles played by NMDAR subtypes.

## 6 Pharmacological investigations into GluN2 subtype-specific plasticity

It is now 30 years since the demonstration of the requirement for NMDAR activation for the induction of CA3-CA1 hippocampal long-term potentiation (LTP; Collingridge et al., 1983) but an on-going focus of considerable interest surrounds the possibility that GluN2 subtypes contribute differentially to synaptic plasticity. Pharmacological tools showing subunit-selectivity are in theory ideal to test hypotheses centred on this area. Early studies employed GluN2B-selective antagonists to show that GluN2B-containing NMDARs were important for the induction of hippocampal and perirhinal long-term depression (LTD) respectively but were not essential for LTP (Liu et al., 2004; Massey et al., 2004). Their additional conclusions that GluN2A-containing NMDARs were alone critical for the induction of LTP have since been tempered somewhat in the light of the relative non-selectivity of NVP-AAM077 at the high concentrations used. Instead, it seems that both subunits can contribute to LTP.

For example, Winder and co-workers showed that NMDAR-dependent hippocampal CA3-CA1 LTP induced by high frequency stimulation did not have an absolute requirement for GluN2A-containing NMDARs, as evidenced by studying GluN2A-deficient slices (Weitlauf et al., 2005). Moreover, they showed that the concentration of the GluN2A-preferring drug NVP-AAM077 (400 nM) used previously to implicate GluN2A-containing NMDARs (Liu et al., 2004) was not selective since it even blocked LTP in GluN2A-deficient slices. The careful use and characterization of low-dose NVP NVP-AAM077 as a GluN2A-preferring antagonist, in combination with GluN2B-selective antagonists also supported a role for both GluN2A and GluN2B in mediating LTP (Bartlett et al., 2007). However, similar studies have concluded that LTP is preferentially induced by GluN2A-containing NMDARs (Moult and Harvey, 2011). Interestingly, Kohr and co-workers used both low-dose GluN2B and GluN2A-preferring antagonists, and genetic loss of GluN2A to conclude that, at least in the case of low frequency stimulation protocols (paired with post-synaptic depolarization) that both GluN2A and GluN2B-containing NMDARs contributed to LTP and that charge transfer/Ca^2+^ influx was the major determining factor rather than participation of any particular subtype (Berberich et al., 2007, 2005). Related to this is the recent observation that at amygdala synapses LTP is mediated via GluN1/GluN2A/GluN2B tri-heteromeric NMDARs (Delaney et al., 2012).

In addition to there being impaired LTP in the GluN2A knockout mouse (Sakimura et al., 1995), genetic evidence also now points to a role for GluN2B: analysis of a mouse containing a forebrain-specific deletion of GluN2B revealed a deficit in paired protocol CA3-CA1 LTP attributable to reduced charge transfer (von Engelhardt et al., 2008). In contrast, field LTP remained unaltered, perhaps reflecting a stronger stimulation that could elicit sufficient NMDAR-dependent Ca^2+^ influx even through the GluN2B-deficient synapses (von Engelhardt et al., 2008). Thus, a tentative consensus is emerging that both GluN2A and GluN2B-containing NMDARs can both contribute to LTP in relatively mature hippocampal slices. However, interpretation of pharmacological experiments is clouded a little by the fact that, as noted above, tri-heteromeric NMDARs containing GluN2A and GluN2B contribute a substantial amount to synaptic NMDAR currents at the CA1 synapse. Moreover, as indicated above, the effect of NVP-AAM077 on these channels, alone or in combination with GluN2B-preferring antagonists (as employed in many studies) is not clear. Also, as noted above, GluN2B-preferring antagonists have limited effects at GluN1/GluN2A/GluN2B triheteromeric receptors, meaning that just because LTP is not blocked by a GluN2B-preferring antagonist, this does not mean that GluN2B is not needed for LTP (it may be playing a critical role as part of GluN1/GluN2A/GluN2B triheteromeric receptors). Also, as noted above, the dose-dependent potency and dose-dependent subunit-selectivity of NVP-AAM077 under non-steady-state conditions of synaptic transmission are less than clear. These issues should be borne in mind when interpreting investigations into GluN2 subtype-specific function based purely on pharmacological tools.

## 7 Use of chimeric GluN2 subunits to probe subunit-specific function

Mammalian GluN2 subunits have large cytoplasmic C-terminal domains (CTDs) that are responsible, along with the CTD of GluN1, for linking the NMDAR to an array of signalling and scaffolding proteins that together form the NMDAR signalling complex (Husi et al., 2000; Kim and Sheng, 2004; Ryan et al., 2008). The amino acid sequences of the different GluN2 subunit CTDs have diverged substantially during evolution, and have the potential to differentially link to signalling molecules (Ryan et al., 2008). This led to the hypothesis that in addition to the GluN2 subtype conferring specific biophysical properties on the NMDAR, the GluN2 CTD subtype may additionally provide functional diversity by influencing downstream effectors of NMDAR activation. This has been investigated recently in the context of synaptogenesis, synaptic plasticity, excitotoxicity, and behaviour and cognition, with a focus on differential effects of the CTDs of GluN2A and GluN2B, the adult major forebrain GluN2s.

An important requisite of such studies is to uncouple any influence of the channel/ligand-binding portion of the GluN2 subunit from that of the C-terminus. An approach taken by several labs has been to investigate the functional consequences of the expression of both wild-type GluN2A and GluN2B compared to expression of chimeric GluN2 subunits in which the CTD of one subunit has been switched with that of the other. By studying, for example, the consequence of GluN2B expression compared with that of expressing GluN2B with its CTD replaced with that of GluN2A (and *vice-versa*), the effects of the CTD subtype can be investigated in isolation. These approaches are valid because there is no evidence that swapping the GluN2 CTD subtype would influence the gating properties of the NMDAR. Indeed, this possibility has now been directly tested and ruled out (Maki et al., 2012; Punnakkal et al., 2012).

Using this approach Sheng and co-workers demonstrated an important role for the GluN2B CTD in hippocampal LTP: knockdown of GluN2B inhibited LTP and could be rescued by expression of a RNAi-resistant form of GluN2B but not by GluN2A or by GluNB with its CTD replaced by that of GluN2A (Foster et al., 2010). Conceptually similar approaches were employed by Gambrill and Barria to investigate GluN2 subtype-specific synaptogenesis and stabilization (Gambrill and Barria, 2011). Premature GluN2A over-expression resulted in lowered spine density and miniature EPSC frequency in the hippocampus, indicative of restricted synaptogenesis. The effect could be recapitulated by over-expressing GluN2B with the CTD of GluN2A, but not by wild-type GluN2B nor by GluN2A with the CTD of GluN2B, thus implicating the GluN2A CTD specifically in this phenomenon. Further experiments implicated the reported inability of CaMKII to interact with the GluN2A CTD as being responsible for this, suggestive of a specific role for the GluN2B CTD in normal synaptic development.

Grant, Komiyama and co-workers recently used an alternative, and elegant, approach which enabled them to study the role of GluN2 CTD subtype in the whole animal (Ryan et al., 2013). Rather than ectopically expressing chimeric GluN2 subunits, they made two knock-in mice by targeted exon exchange: one in which the CTD of GluN2B was replaced with that of GluN2A, and one in which the CTD of GluN2A was replaced with that of GluN2B. Strikingly, analysis of a battery of learnt and innate behaviours in these mice revealed differing requirements for the two GluN2 CTDs. Some tasks were insensitive to either replacement of the CTD of GluN2A (with that or GluN2B) or the replacement of the CTD of GluN2B (with that of GluN2A). In contrast, several tasks had an absolute requirement for the CTD of GluN2B only, one task had an absolute requirement of the CTD of GluN2A, and one appeared to require the presence of both CTDs. Parallel investigations were also performed aimed at investigating the role of GluN2 CTD subtype in hippocampal synaptic plasticity. Using both theta-burst and theta-pulse induction of LTP it was shown that the GluN2A CTD could be swapped for that of GluN2B without influencing plasticity. In contrast, exchange of the CTD of GluN2B for that of GluN2A enhanced theta-burst LTP but suppressed theta-pulse LTP. This places the GluN2B CTD as being important for decoding specific patterns of electrical activity in a manner that cannot be achieved by the GluN2A CTD. Intriguingly, mice expressing GluN2B with the CTD of GluN2A exhibited far lower levels of interaction between GluN2B and the MAGUK proteins PSD-95 and PSD-93, suggesting that the GluN2B CTD may link to a qualitatively or quantitatively different protein complex in the forebrain. In potential support of this idea is the recent observation that GluN2A-containing NMDARs induce LTP via a mechanism dependent on Ras-GRF2, while GluN2B-containing NMDARs induce LTP independent of this pathway (Jin and Feig, 2010). Collectively these data support the notion that ancestral duplication of the GluN2 gene has enabled sequence divergence, leading to increased diversity in synaptic signalling, potentially underlying the capacity for an enhanced repertoire of complex behaviours (Ryan et al., 2013).

**Fig. 7:**
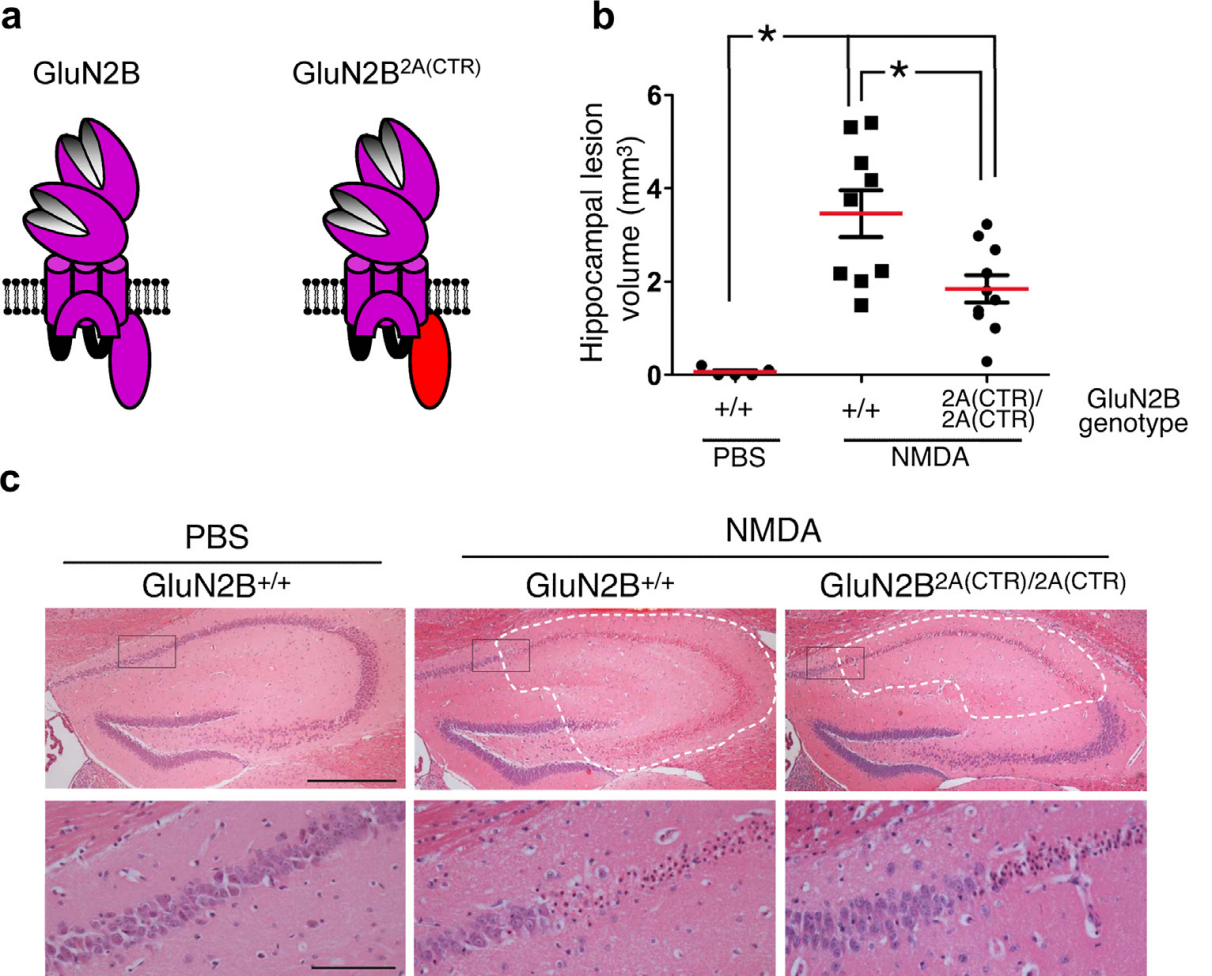
The identity of the GluN2 CTD determines the response to excitotoxic insult. (a) Cartoon depiction illustrating the C-terminal replacement (CTR) of the GluN2B CTD with that from GluN2A and denoted as GluN2B^2A(CTR)^. (b) Quantification of hippocampal lesion volumes 24 h after stereotaxic injection of phosphate buffered saline (PBS) or 15 nmol of NMDA into the hippocampi of either GluN2B^+/+^ or GluN2B^2A(CTR)/2A(CTR)^ mice. Note the significant reduction in lesion volumes when the GluN2B CTD is replaced by that of GluN2A. (c) Upper panels, representative pictures of haematoxylin and eosin-stained hippocampal sections showing the extent of NMDA-induced damage (dashed lines); lower panels, higher magnification of the boxed areas shown in the upper panels. Calibration bars in upper and lower panels are 250 μm and 50 μm, respectively. Panels (b) and (c) from Martel et al. (2012). Copyright © 2012 Cell Press. Used with permission.

We recently addressed the role of GluN2 CTD subtype in determining NMDAR excitotoxicity (Martel et al., 2012). Ectopic expression of chimeric GluN2A and 2B subunits with reciprocal exchanges of their CTDs showed that compared to the CTD of GluN2A that of GluN2B more efficiently coupled NMDAR-dependent Ca^2+^ influx to neuronal death. Indeed, this was observed regardless of whether the GluN2B was attached to the rest of the GluN2B subunit, or as part of a chimeric subunit linked to GluN2A. In collaboration with Grant and Komiyama we studied excitotoxicity *in vitro* and *in vivo* utilizing their mouse expressing GluN2B with its CTD replaced with that of GluN2A. In exact agreement with our ectopic expression studies, replacing GluN2B's CTD with that of GluN2A reduced neuronal sensitivity to NMDAR-dependent Ca^2+^ influx both *in vitro* culture and *in vivo* following an excitotoxic insult (Fig. 7). The molecular basis for this GluN2 CTD subtype-specific effect was an increased physical and functional coupling of the GluN2B CTD to a PSD-95-nNOS-dependent CREB dephosphorylation pro-death pathway, although contributions from other CTD subtype-specific pathways are likely to exist and await further investigation. It should be noted that not all GluN2B-mediated signalling is bad though – activation of synaptic GluN2B-containing NMDARs promoted by physiological action potential bursting is potently neuroprotective (Al-Mubarak et al., 2009; Martel et al., 2009b; Papadia et al., 2008). The toxic consequences of GluN2B CTD signalling are only observed in the context of chronic activation of all (synaptic and extrasynaptic NMDARs) by elevated ambient levels of agonist (Martel et al., 2009b). Under these conditions the chronic, tonic activation of NMDARs, particularly extrasynaptic NMDARs leads to very different downstream effects in terms of signalling cascades (Hardingham and Bading, 2002; Ivanov et al., 2006; Leveille et al., 2008; Papadia et al., 2008) and transcriptional outputs (Hardingham and Bading, 2002; Soriano et al., 2009; Wahl et al., 2009; Zhang et al., 2007) with deleterious consequences (for a comprehensive review on this topic see Hardingham and Bading (2010)).

It will also be important to determine whether GluN2 CTD subtype determines the progression or severity of neurological conditions associated with abnormal or inappropriate NMDAR function, such as ischaemia, traumatic brain injury or chronic progressive disorders such as Huntington's disease. Neuroprotective peptide mimetics of the GluNB CTD and downstream interacting proteins are an emerging area of interest, with several showing promise in rodent stroke models (Aarts et al., 2002; Bach et al., 2012; Tu et al., 2010). Particularly exciting is a cell-permeable peptide mimetic of the GluN2B PDZ ligand (NA-1, Tat-NR2B9c (Aarts et al., 2002)). This peptide was found to partly uncouple GluN2B-containing NMDARs from PSD95, reducing NO production following excitotoxic insult and suppressing pro-death p38 signalling and CREB shut-off (Aarts et al., 2002; Martel et al., 2012; Soriano et al., 2008). Also, unlike conventional NMDAR antagonists, NA-1/Tat-NR2B9c did not interfere with activity-dependent synaptic potentiation, nor synaptic NMDAR-dependent neuroprotective signalling via Akt or CREB (Martel et al., 2009a; Soriano et al., 2008).

Moreover NA-1/Tat-NR2B9c was shown to be effective post-treatment in reducing lesion size and improving outcome following stroke in the non-human primate brain (Cook et al., 2012). NA-1 has also advanced to human clinical trials for iatrogenic stroke after endovascular aneurysm repair (Hill et al., 2012). Phase 2 results published very recently revealed no adverse side effects to NA-1, and a significant reduction in the number of ischemic infarcts compared to placebo (Hill et al., 2012). To conclude, the idea that GluN2B CTD-specific signalling underlies neuronal dysfunction and death in a variety of neurological conditions is an attractive one, and although the role of the GluN2B CTD in plasticity and behaviour is becoming clear, recent work has shown that it is nevertheless realistic to hope that targeting the CTD will give far less severe side effects than global antagonists.

## 8 Conclusions

In this review we have focussed on the role played by the GluN2 subunit in controlling NMDAR function. These subunits are critical determinants of NMDAR heterogeneity and provide the major influence in determining NMDAR subtype biophysical, pharmacological and signalling properties. In recent years our rapidly advancing understanding of NMDARs has allowed for the identification of structural elements and, at times, single amino acid residues, which determine GluN2-specific control of function. This understanding provides us with opportunities to develop novel pharmacological agents with which we can probe NMDAR subtype-specific physiological and pathophysiological functions, the ultimate aim of which is the treatment of neurological dysfunction. Indeed, as indicated in the final section of the review selective targeting of GluN2-dependent function shows such strategies possess considerable clinical promise.
